# Severe Gastrointestinal Toxicity Following the Use of Gilteritinib: A Case Series and Analysis of Postmarketing Surveillance Data

**DOI:** 10.3390/healthcare11101479

**Published:** 2023-05-18

**Authors:** Lucia Gozzo, Antonella Nardo, Serena Brancati, Antongiulio Judica, Andrea Duminuco, Cinzia Maugeri, Marina Parisi, Laura Longo, Daniela Cristina Vitale, Rosy Ruscica, Giovanni Luca Romano, Elisa Mauro, Paolo Fabio Fiumara, Giuseppe Alberto Maria Palumbo, Francesco Di Raimondo, Calogero Vetro, Filippo Drago

**Affiliations:** 1 Clinical Pharmacology Unit, Regional Pharmacovigilance Centre, Azienda Ospedaliero Universitaria Policlinico “G. Rodolico—S. Marco”, 95123 Catania, Italy; longolaura@hotmail.com (L.L.); danielac.vitale@gmail.com (D.C.V.); rosyruscica@gmail.com (R.R.); f.drago@unict.it (F.D.); 2 Haematology Unit, Azienda Ospedaliero Universitaria Policlinico “G. Rodolico—S. Marco”, 95123 Catania, Italy; antonella.nardo@live.it (A.N.); andrea.duminuco@gmail.com (A.D.); maugericinzia@hotmail.com (C.M.); marinaparisi@hotmail.it (M.P.); elixmauro@hotmail.it (E.M.); paolo.fiumara@virgilio.it (P.F.F.); palumbo.giuseppealberto@gmail.com (G.A.M.P.); diraimon@unict.it (F.D.R.); gerovetro@gmail.com (C.V.); 3 Gastroenterology Unit, Azienda Ospedaliero Universitaria Policlinico “G. Rodolico—S. Marco”, 95123 Catania, Italy; antongiuliojudica@gmail.com; 4 Department of Biomedical and Biotechnological Sciences, University of Catania, 95123 Catania, Italy; giovanniluca.romano@unict.it; 5 Department of Scienze Mediche Chirurgiche e Tecnologie Avanzate “G.F. Ingrassia”, University of Catania, 95123 Catania, Italy; 6 Department of Chirurgia Generale e Specialità Medico-Chirurgiche, University of Catania, 95123 Catania, Italy; 7 Centre for Research and Consultancy in HTA and Drug Regulatory Affairs (CERD), University of Catania, 95123 Catania, Italy

**Keywords:** gilteritinib, acute myeloid leukemia, adverse events, serious gastrointestinal disorders, risk management plan

## Abstract

Gilteritinib has been approved as monotherapy in adults with acute myeloid leukemia (AML) FLT3 mutated with relapsed or refractory disease, in light of its advantages in terms of survival and the favorable safety profile. Hepatobiliary disorders and musculoskeletal and connective tissue disorders represent the most frequent adverse reactions associated with gilteritinib, whereas the most frequent serious adverse reaction is acute kidney injury. In the summary of product characteristics, gastrointestinal (GI) events are indicated as very common, in particular diarrhea, nausea and stypsis. Furthermore, serious GI disorders have been observed with gilteritinib in clinical trials, including GI hemorrhage, GI perforation and GI obstruction. However, the association with the FLT3 inhibitor has not been confirmed. Nevertheless, serious GI AEs have been recognized as an important potential risk to be monitored in postmarketing surveillance. We present three cases of serious self-limiting GI events observed in patients on gilteritinib treatment for AML, and an analysis of relevant available postmarketing surveillance data.

## 1. Introduction

FMS-like tyrosine kinase 3 (FLT3) inhibitors represent the new standard of care for patients with *FLT3*-mutant acute myeloid leukemia (AML), in both the first line and salvage settings [1,2,3,4,5]. First-generation FLT3 multitargeted tyrosine kinase inhibitors (TKIs), such as midostaurin and sorafenib, are limited by poor drug selectivity, weak potency and unfavorable protein-binding characteristics [6]. Second- and third-generation FLT3 inhibitors have improved selectivity and potency resulting in much higher clinical response rates and a favorable safety profile [7,8]. 

Gilteritinib was approved in Europe in 2019 as monotherapy for AML with an *FLT3* mutation in adults with relapsed or refractory disease [9,10,11,12,13,14,15,16,17].

The results from clinical trials showed an advantage in terms of response rate and overall survival (OS) with gilteritinib monotherapy compared to salvage chemotherapy [10,11]. Moreover, gilteritinib treatment was associated with a good safety profile, resulting in a low incidence of grade ≥ 3 adverse events (AEs) [10,18]. The most frequent adverse reactions with gilteritinib are hepatobiliary disorders and musculoskeletal and connective tissue disorders, whereas the most frequent serious adverse reaction is acute kidney injury [19].

Serious gastrointestinal (GI) disorders have been observed in patients treated with gilteritinib in clinical trials, including GI hemorrhage, GI perforation and GI obstruction [9]. However, the association with the FLT3 inhibitor has not been confirmed. Nevertheless, serious GI AEs have been recognized as an important potential risk in the risk management plan (RMP) to be monitored in postmarketing surveillance. The drug is under so-called ‘additional monitoring’ by the European Medicines Agency (EMA); thus, it is monitored more intensively than other medicines to allow for a rapid identification of new safety information. 

In this paper, we present three cases of serious self-limiting GI events observed in patients on gilteritinib treatment for AML and an analysis of updated relevant postmarketing surveillance data.

## 2. Materials and Methods

A retrospective analysis of AEs observed in patients treated with gilteritinib was performed at the Haematology Unit of the University Hospital of Catania from June 2019 to December 2022. Data about clinical history, comorbidities and treatment details were collected with a specific focus on GI adverse drug reactions (ADRs) onset and recovery, seriousness, outcome, dechallenge, rechallenge, relevant laboratory tests or diagnostic procedures and concomitant drugs. For each case, a causality assessment was performed according to the Dx3 method [20]. This approach allows us to qualitatively assess the relationship between the use of a medicine and AEs using a checklist to guide the analysis of the following three domains: the drug disposition, the pre-disposition of the patient (vulnerability) and the disposition of the patient–drug interaction (mutuality).

Moreover, a pharmacovigilance analysis was performed using data reported on the website (www.adrreports.eu) of suspected ADRs of the European pharmacovigilance database (EudraVigilance, EV) before 31 December 2022. We analyzed all the individual case safety reports (ICSRs) related to gilteritinib, focusing on ICSRs reporting GI events. Suspected ADRs were grouped according to the Medical Dictionary for Regulatory Activities (MedDRA^®^) [5,21,22,23,24] and defined as serious if they were life-threatening or fatal, required hospitalization (or prolonged existing hospitalization), resulted in persistent or significant disability, or represented a congenital anomaly/birth defect or other medically important condition [25]. 

Descriptive statistics were used to summarize data, reporting frequencies and percentages for categorical data and median values for continuous data.

## 3. Results

In the reference period, a total of 19 patients were treated with gilteritinib at the Haematology Unit of the University Hospital of Catania. Three of them (15%) showed a self-resolved Grade 3 (G3) GI toxicity, including melena and rectorrhagia. 

### 3.1. Case 1 Presentation

The first case concerns a 38-year-old female patient who came to our attention for the sudden onset of severe fever. The complete blood count showed mild platelet deficiency (platelets (plt) 113,000/uL), anemia (hemoglobin (hb) 8.5 g/dL) and hyperleukocytosis (white blood cells (WBC) 210,000/uL). Bone marrow (BM) aspiration and sampling for flow-cytometry, molecular biology and cytogenetics were performed. A population of 22% immature blast cells CD117+, CD38+, HLA-DR+ and CD45+ was found. Karyotype was normal (46, XX). The *NPM1* gene was mutated, as well as *IDH1* (R132H) and *FLT3*-ITD with a very low allelic ratio (AR), i.e., 0.01. Therefore, based on the ELN2017 classification, a diagnosis of AML with *NPM1* mutation was made, and the patient was classified as low risk. For this reason, the patient underwent induction chemotherapy with the “7 + 3” scheme, based on cytarabine 100 mg/m^2^ intravenous (iv) continuous infusion over 24 h on days 1–7 and daunorubicin 60 mg/m^2^ on days 1, 3 and 5, plus midostaurin (50 mg per day, from day +8 to +21). On day +37, at the BM reevaluation, the patient achieved complete remission (CR) with negativity at minimal residual disease (MRD), documented by the absence of the *NPM1* transcript at quantitative PCR and clearance of the *FLT3*-ITD mutation. Three consolidation cycles, performed with high dose cytarabine (3 g/m^2^ every 12 h on days 1, 3 and 5) (HiDAC) and midostaurin (50 mg per day, from day +8 to +21), maintained the achieved CR with negativity at MRD. These therapies were completed without notable complications. After three months of follow-ups, from the end of the first-line treatment, a BM reevaluation with flow cytometry was performed, revealing a blast cell population equal to 82%, suggestive of AML relapse. In addition, *FLT3*-ITD and *NPM1* mutations were once again detectable. Because of the presence of the *FLT3*-ITD mutation, a second-line treatment with gilteritinib was started at an initial dose of 120 mg once daily. 

Thirty days after gilteritinib treatment was started, the patient experienced gastrointestinal and hemorrhagic adverse events (AEs) such as diarrhea, nausea and rectorrhagia; blood analysis showed severe pancytopenia with hb 7.7 g/dL, PLT 31,000/µL and WBC 790/µL.

Therefore, she was hospitalized and received packed red blood cells (PRBCs) on average one unit every two days throughout the hospitalization, but no PLT transfusions. After two weeks, hemorrhagic and gastrointestinal symptoms resolved without drug interruption, and she was re-evaluated with bone marrow aspiration (BMA) showing blast cells (BC) equal to 2% at morphology with persistence of the *FLT3*-ITD AR:0.3 at molecular biology. In addition, after two months, during further hospitalization, the patient suffered from carbapenemase-producing Klebsiella pneumoniae (KPC) bacteremia, leading to a worsening clinical condition, until multi-organ failure (MOF). The patient died four months after gilteritinib treatment was started due to respiratory failure, despite hospitalization in an intensive care unit and maintaining a state of complete remission.

### 3.2. Case 2 Presentation

**Figure 1:**
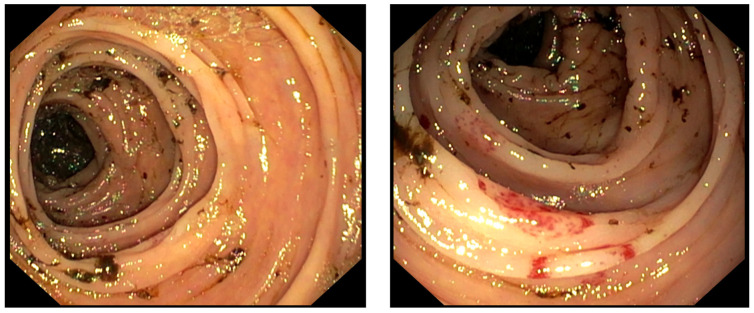
Colonoscopy examination of patient with GI toxicity related to gilteritinib, showing evidence of ulcerated lesions localized in the descending colon.

The second case concerns a 65-year-old patient diagnosed with AML which developed after myelodysplastic syndrome (MDS) and was treated with the hypomethylating agent azacytidine for a total of 26 cycles. At the end of the 26th cycle, a compatible donor was found, and the patient was addressed to a BM transplant. During the transplant eligibility work-up, unfortunately, the patient experienced an abscess in the left parotid region, and treatment with large broad antibiotics was started. During the antibiotic therapy, a bone marrow re-evaluation was performed showing, at morphological examination, about 50% of myeloid blasts, confirmed by flow-cytometry. A few days later, the patient experienced hyperchromic (purplish-red) and non-itchy nodular lesions in the chest and abdomen, highly suggestive of extramedullary involvement of AML. For this AML relapse, he underwent treatment with azacytidine plus venetoclax (AZA-VEN). Upon re-evaluation, performed after the fifth cycle, the count of blast cells was about 10%. For the first time, he reported the onset of *FLT3*-ITD mutation. Considering the poor response to AZA-VEN therapy, the patient stopped the treatment and began gilteritinib therapy at 120 mg per day. On day +29, morphological re-evaluation of the disease showed an absence of response (i.e., blast cells 13%). Therefore, the drug dose was increased to 200 mg per day. At day +45, complete remission of the disease was morphologically assessed. During the surveillance period, however, the patient reported GI clinical toxicity manifested with abdominal pain, diarrhea and melena associated with hematological toxicity resulting in severe pancytopenia (hb 6.8 g/dL; PLT 4000/µL; WBC 2620/µL; Neutrophils (N) 1240/µL). Coagulative test results were abnormal, with an increased international normalized ratio (INR) and D-dimer values (INR 1.43; D-dimer 3006). For this complication, the patient was hospitalized in our Internal Medicine Unit, where he received transfusion support of platelets (PLT) and PRBC daily and underwent a colonoscopy examination showing evidence of ulcerated lesions localized in the descending colon (Figure 1). On histological examination, it was described as “colonic mucosa focally eroded, lined with fibrinous induction, with moderate lymphoplasmacytic infiltrate in the lamina propria; absence of cryptitis and micro-abscesses; glandular architecture and muciparous activity are maintained”. The treatment with gilteritinib was not interrupted, despite the adverse reaction, and the bleeding resolved in about a week. At the morphological re-evaluation with bone marrow aspirate examination (BMA), three months after starting gilteritinib, he was in complete remission. Unfortunately, a few days later, the patient eventually died due to septic neutropenic fever.

### 3.3. Case 3 Presentation

The last case concerns a 68-year-old man suffering from therapy-related AML. The patient had received a previous chemotherapy treatment, according to the scheme VACOP-B, for a diffuse large B-cell lymphoma (DLBCL) that was still in remission at the time of AML diagnosis. At AML presentation, he displayed anemia (Hb 9.7 g/dL), thrombocytopenia (PLT 50,000/uL) and hyperleukocytosis (WBC 90,000/uL). A BM evaluation was performed, confirming the diagnosis of AML. At molecular biology, *NPM1* was non-mutated and *FLT3*-ITD was negative; karyotype was normal. Therefore, he underwent chemotherapy with liposomal daunorubicin/cytarabine (CPX351) for two cycles, maintaining a stable disease. Considering the chemorefractoriness, we decided to shift to a second-line therapy with AZA-VEN, achieving only a slight reduction in the amount of blast cells (from 40% to 20%) in the bone marrow after two cycles of therapy. At disease re-evaluation, *FLT3*-ITD was positive. Therefore, a third-line therapy with gilteritinib was started at a dose of 120 mg per day. At the re-evaluation, 30 days after gilteritinib treatment was started, the patient was in CR. Unfortunately, during the preparatory work-up for bone marrow transplantation, the patient was hospitalized elsewhere because of a SARS-CoV-2 infection and was discharged about a month later with a negative swab. During hospitalization, treatment with gilteritinib was continued. At this point, the patient was re-evaluated, on day +90 from the start of the gilteritinib therapy. At morphological evaluation, an increase in blast percentage was seen. Thus, the dose of gilteritinib was increased from 120 to 200 mg per day. Due to the occurrence of febrile neutropenia, he was then hospitalized and treated with specific antibiotic therapy. During hospitalization, the patient developed GI toxicity, resulting in melena (Hb 8; PLT 21,000; WBC 13,190; N 5490). The bleeding resolved in just three days, and the patient was then discharged in good general condition. The patient continued treatment with gilteritinib without changing the dosage. At the most recent disease re-evaluation, flow cytometry showed a percentage of blast cells equal to 27.8% and a monocyte component equal to 2.3%, already present at the onset of AML. Treatment with gilteritinib was pursued because late responders have been described in the literature. At the subsequent follow-up visits, the patient maintained a good clinical condition for about two months. PLT and PRBC transfusions were not necessary. However, in the last month, we witnessed a new worsening of his blood count, up to blood transfusions (Hb 6) but with a platelet count above the transfusion threshold. On day + 200 after starting gilteritinib therapy, the patient came to our attention with a poor general clinical condition and fever. Hematology evaluation showed disease progression with hyperleukocytosis (WBC 147,390/uL). During hospitalization, he developed kidney failure and jaundice. Unfortunately, four days after admission, the patient died due to cerebral hemorrhage.

### 3.4. Causality Assessment

Drug disposition: As previously described, there is evidence for a plausible mechanism by which gilteritinib can induce GI toxicity, coming from both preclinical and clinical data [9]. The GI tract represents a target organ of toxicity in several animal models, showing reversable GI epithelial damage and inflammation. Moreover, GI AEs have been reported in clinical trials and postmarketing surveillance. These findings provide *strong evidence for a disposition* of gilteritinib to the reported events.

Patient vulnerability: None of the three patients had experienced GI events before the administration of gilteritinib, and they did not have risk factors for serious GI disorders in AML, such as sepsis and infection. GI bleeding was observed in all three cases, and this could be related to the low platelet count, secondary to the disease or to the hematological toxicity of the drug. There is *good evidence for the vulnerability* of the patient to the reported events.

Mutuality: The temporal development (all cases) and the dose–response (case 2 and 3) of the events indicate a plausible interaction between patient’s disposition and the drug’s properties. Therefore, there is *strong evidence of drug–patient interaction*.

### 3.5. Data from the EudraVigilance Database 

By 31 December 2022, 1105 ICSRs had been identified on the EudraVigilance Database for gilteritinib (Table 1), in patients mainly belonging to the age groups 65–85 (40.2%) and 18–64 years (38.8%) and to the male sex (48.2% vs. 43.7% female). Of all the ICSRs, 95.6% were classified as serious. The most frequent reactions fall within the group ‘*Investigations*’ (n = 370; 33.5%), followed by ‘*Blood and lymphatic system disorders*’ (n = 355; 32.1%) and ‘*Infections and infestations*’ (n = 282; 25.5%). There were 129 GI disorders (11.7%; Table 2), observed mostly in females (52.7%) in the age group 65–85 years (41.9%); 125 (96.9%) were serious, including 5 fatal events (3.5%; 1 GI perforation, 1 hematemesis, 1 ileus and 2 neutropenic colitis; Table 3). Among the GI events, the most frequent were diarrhea (n = 31) and nausea/vomiting (n = 25/10), followed by gastrointestinal bleeding (gastrointestinal hemorrhage = 9; melena = 6; rectal hemorrhage = 4; diarrhea hemorrhagic = 1; enterocolitis hemorrhagic = 1; gastric hemorrhage = 1; gastritis hemorrhagic = 1; hematemesis = 1; hematochezia = 1; intestinal hemorrhage = 1; intra-abdominal hematoma =1; small intestinal hemorrhage = 1; upper gastrointestinal hemorrhage = 1).

## 4. Discussion

Given the established role of these mutations in AML, FLT3 inhibitors became a standard of care among leukemia therapies. Several competitive inhibitors acting via the ATP-binding sites of the FLT3 receptor have been developed and are currently in clinical trials. First-generation inhibitors (sorafenib and midostaurin) are less specific for FLT3 and have more off-target toxicities; second-generation FLT3 inhibitors, including gilteritinib, are more specific and potent with better safety profiles [26]. Therefore, it is essential to clarify the different safety profiles of these agents to ensure a more individualized treatment adapted to patients’ needs.

The drug is primarily metabolized by CYP3A4, leading to potential drug–drug interactions, in particular with CYP inhibitors, which can increase gilteritinib plasma concentration and the risk of serious AEs [9].

In this context, antifungal prophylaxis deserves special attention due to the metabolic interactions with azole drugs (such as itraconazole, fluconazole and posaconazole, the standard of care used as prophylaxis in AML patients) [27].

In the summary of product characteristics (SmPC) of the product, GI adverse events are indicated as frequent, in particular diarrhea (35.1%, 4.1% as G3 or more), nausea (29.8%, G3 1.9%) or stypsis (28.2%, G3 0.6%). Moreover, the EMA office warned about some cases with GI perforation, bleeding or obstruction. 

The GI tract was recognized as a target organ of toxicity in several animal models: e.g., in rats, microvacuolation of the GI mucosal epithelium was observed, and in dogs, the fecal occult blood reaction was positive and inflammation was found on the alveolus/gingiva of the teeth [9]. In both models, these toxicities occurred at exposures below those at the recommended dose of 120 mg in clinical trials, and most of the effects were reversible by the end of a 4-week recovery period.

Risk factors for serious GI disorders in AML patients include sepsis and infection.

The pivotal trial ADMIRAL (2215-CL0301), a phase III open-label, multicenter, randomized study, demonstrated the advantage of gilteritinib versus salvage chemotherapy in patients with R/R AML with *FLT3* mutation in terms of OS (9.3 months in the gilteritinib group versus 5.6 months in the chemotherapy group; hazard ratio (HR), 0.637; 95% confidence interval (CI), 0.490–0.830; *p* = 0.0004) and the rate of complete remission/complete remission with partial hematologic recovery (CR/CRh) (34% vs. 15.3%, *p* = 0.0001) [10]. The drug at the proposed therapeutic dose of 120 mg demonstrated a good safety profile, with manageable adverse events generally associated with the pathophysiology of the disease and the known toxicity of other inhibitors.

The safety analysis included data from a total of 522 patients enrolled in the study 2215-CL-0101 (n = 252), in the study 2215-CL-0102 (n = 24) and in the study 2215-CL-0301 (n = 246), and who received at least one dose of gilteritinib (median duration of exposure = 111 days, 4–1320 days) [18]. Among these patients, 319 received a starting dose of 120 mg of gilteritinib. Overall, 99.4% of patients experienced at least one treatment-emergent adverse event (TEAE), of which 83.1% were drug-related and 29% warranted dose interruption. The most frequent TEAEs included abnormal laboratory findings (increase in blood creatine phosphokinase, alanine aminotransferase (ALT), aspartate aminotransferase (AST) and alkaline phosphatase) and gastrointestinal (diarrhea, nausea and constipation) and respiratory toxicities (cough and dyspnea). The most frequent grade 3 or higher drug-related TEAEs were hematological toxicities (anemia, febrile neutropenia and thrombocytopenia).

These results are in line with our findings on the EudraVigilance database, where ‘*Investigations*’ and ‘*Blood and lymphatic system disorders*’ represent the most frequent events in postmarketing surveillance. 

In general, AEs that arise from gilteritinib therapy are manageable based on treatment interruptions or dose reductions [28].

In the 450 mg dose-escalation cohort, diarrhea represented one of the two Dose-Limiting Toxicities (DLTs) [18]. Grade ≥ 3 diarrhea occurred in the 120 mg/d (n = 1) and 200 mg/d (n = 2) dose groups. GI bleeding was already reported in the phase 1/2 study, 1.6% as G3 AE, 0.4% as G4 AE. In the paper by Perl et al., intestinal perforation was reported in 0.8% of cases (i.e., two patients) [10]. 

In patients who underwent BM transplantation and resumed gilteritinib, the most frequent AEs included diarrhea (40%) and one case of fatal acute GI graft-versus-host disease (GVHD) [29].

A postmarketing study in Japan investigated the efficacy and safety of gilteritinib + azacitidine versus azacitidine in adults with newly diagnosed *FLT3* + AML. In patients unfit for intensive chemotherapy, the CR rate was higher with gilteritinib + azacitidine than with azacitidine [30]. GI events were among the most common AEs observed with gilteritinib + azacitidine (diarrhea 38.4%, constipation 34.2% and nausea 32.9%), with a higher incidence compared to azacitidine alone. GI hemorrhage occurred in 12.3% of patients (n = 9) in the gilteritinib + azacitidine group: 15 episodes were TEAEs, 10 resolved and 11 were not considered related to gilteritinib. In the three cases, the GI hemorrhage occurred in proximity to patient death, and in two cases, before treatment discontinuation, suggesting the possibility of the onset of these events in the context of AML progression. The administration of gilteritinib + azacitidine led to the onset of grade ≥3 treatment-related GI hemorrhages in 5.5% of patients (n = 4), none of which had a fatal outcome. 

In line with preclinical and clinical evidence, we observed three cases of GI toxicities which required hospitalization and diagnostic/therapeutic procedures, but all were self-limiting without drug interruption. Similarly, the GI events reported on EudraVigilance were almost all serious, recovered or recovering in the majority of cases with a known outcome.

Those with fatal outcomes were all associated with other serious ADRs, including infections, sepsis and differentiation syndrome, and three out of five were related to the use of gilteritinib with other suspected drugs.

## 5. Conclusions

In conclusion, these data support the knowledge about the GI toxicities identified in nonclinical and clinical studies with gilteritinib.

More studies need to be carried out in the future in order to understand the exact role of FLT3 inhibition in target organs, as well as the possible increased risk when in association with other drugs.

## Figures and Tables

**Table 1:** Individual cases identified in EudraVigilance for gilteritinib before 31 December 2022.

Variable	Cases	%
**Reporter group**		
Healthcare Professional	1061	96.0
Non-Healthcare Professional	44	4.0
Not Specified	/	/
**Age group**		
Not Specified	148	13.4
0–1 Month	/	/
2 Months–2 Years	/	/
3–11 Years	10	0.9
12–17 Years	12	1.1
18–64 Years	429	38.8
65–85 Years	444	40.2
More than 85 Years	62	5.6
**Sex**		
Female	483	43.7
Male	533	48.2
Not Specified	89	8.1
**Seriousness**		
Non-serious	49	4.4
Serious	1056	95.6
**Reaction Groups**		
Blood and lymphatic system disorders	355	32.1
Cardiac disorders	60	5.4
Congenital, familial and genetic disorders	14	1.3
Ear and labyrinth disorders	2	0.2
Endocrine disorders	6	0.5
Eye disorders	9	0.8
Gastrointestinal disorders	129	11.7
General disorders and administration site conditions	273	24.7
Hepatobiliary disorders	137	12.4
Immune system disorders	67	6.1
Infections and infestations	282	25.5
Injury, poisoning and procedural complications	162	14.7
Investigations	370	33.5
Metabolism and nutrition disorders	48	4.3
Musculoskeletal and connective tissue disorders	35	3.2
Neoplasms benign, malignant and unspecified (incl cysts and polyps)	268	24.3
Nervous system disorders	79	7.1
Pregnancy, puerperium and perinatal conditions	/	/
Product issues	4	0.4
Psychiatric disorders	11	1.0
Renal and urinary disorders	65	5.9
Reproductive system and breast disorders	3	0.3
Respiratory, thoracic and mediastinal disorders	88	8.0
Skin and subcutaneous tissue disorders	76	6.9
Social circumstances	2	0.2
Surgical and medical procedures	4	0.4
Vascular disorders	34	3.1

**Table 2:** Individual cases of the reaction group ‘gastrointestinal disorders’ identified in EudraVigilance for gilteritinib before 31 December 2022.

**Variable**	Cases	%
**Reporter group**		
Healthcare Professional	121	93.8
Non-Healthcare Professional	8	6.2
Not Specified	/	/
**Age group**		
Not Specified	18	14.0
0–1 Month	/	/
2 Months–2 Years	/	/
3–11 Years	1	0.8
12–17 Years	2	1.6
18–64 Years	51	39.5
65–85 Years	54	41.9
More than 85 Years	3	2.3
**Sex**		
Female	68	52.7
Male	60	46.5
Not Specified	1	0.8
**Seriousness**		
Non-serious	4	3.1
Serious	125	96.9
**Outcome**		
Fatal	5	3.5
Not Recovered/ Not Resolved	13	8.2
Not Specified	/	/
Recovered/ Resolved	41	28.9
Recovered/ Resolved with Sequelae	/	/
Recovering/ Resolving	20	14.1
Unknown	63	44.4

**Table 3:** Individual cases of the reaction group ‘gastrointestinal disorders’ with fatal outcomes.

Case	Age	Sex	Suspect	Duration	Action Taken	Reactions	Duration	Outcome	Seriousness
**1**	18–64	F	Gilteritinib 120 mg	>4000 days	Drug withdrawn	Cognitive deterioration	**/**	Unknown	Hospitalization
						Pharyngotonsillitis	**/**	Fatal	Death Hospitalization
						Bilateral pneumonia	**/**	Fatal	Death Hospitalization
						Acute respiratory failure	**/**	Fatal	Death Hospitalization
						Differentiation syndrome	**/**	Fatal	Death Hospitalization
						Malnutrition	**/**	Unknown	Hospitalization
						*Neutropenic enterocolitis*	**/**	Fatal	Death Hospitalization
						Sepsis	**/**	Fatal	Death Hospitalization
						General health deterioration	**/**	Unknown	Hospitalization
**2**	18–64	F	Gilteritinib 200 mg	/	Not applicable	Fungemia	14 days	Recovered/ Resolved	Death Life threatening Hospitalization
			Cytarabine 240 mg	/	Not applicable	Febrile neutropenia	43 days	Recovered/ Resolved	Death Life threatening Hospitalization
						Respiratory failure	43 days	Recovered/ Resolved	Death Life threatening Hospitalization
			Idarubicin 29 mg	/	Not applicable	Acute kidney injury	37 days	Recovered/ Resolved	Death Life threatening Hospitalization
						*Neutropenic enterocolitis*	/	Fatal	Death Life threatening Hospitalization
**3**	65–85	F	Gilteritinib 120 mg	25 days	Not applicable	*Hematemesis*	/	Fatal	Death
						Infection *P. aeruginosa*	/	Fatal	Death
						Pseudomonal sepsis	/	Fatal	Death
						Mucositis	/	Fatal	Death
**4**	65–85	M	Gilteritinib 120 mg	51 days	Drug withdrawn	AST increased	/	Not Recovered/ Not Resolved	/
						Pyrexia	7 days	Recovered/ Resolved	/
						*Ileus*	/	Fatal	Death
						Respiratory failure	/	Fatal	Death
			Gilteritinib 80 mg	60 days	Drug withdrawn	Liver function test increased	/	Not Recovered/ Not Resolved	/
						Pneumonia	/	Fatal	Death
						ALT increased	/	Not Recovered/ Not Resolved	/
						LDH increased	/	Recovering/ Resolving	/
			Ampicillin-sulbactam	6 days	/	Pneumonia	20 days	Recovered/ Resolved	Hospitalization
						Neutrophil count decreased	129 days	Recovered/ Resolved	Hospitalization
						Enterocolitis	/	Not Recovered/ Not Resolved	Hospitalization
**5**	18–64	M	Gilteritinib	33 days	Not applicable	*Clostridium colitis*	/	Fatal	Death Hospitalization
						Septic shock	/	Fatal	Death Hospitalization
						*Gastrointestinal perforation*	/	Fatal	Death Hospitalization
						Aplasia NOS	/	Unknown	Other

## Data Availability

The datasets for this article are not publicly available because they contain sensitive data of patients. Requests to access the datasets should be directed to the corresponding author.
